# Bile acid receptors regulate the role of intestinal macrophages in inflammatory bowel disease

**DOI:** 10.3389/fimmu.2025.1577000

**Published:** 2025-06-17

**Authors:** Hongjie Yang, Xianhong Shi, Beibei Wang, Heng Li, Bin Li, Tianqi Zhou, Lulu Tian, Shengjun Wang, Kai Yin

**Affiliations:** ^1^ Department of General Surgery, Affiliated Hospital of Jiangsu University, Jiangsu University, Zhenjiang, Jiangsu, China; ^2^ Department of Laboratory Medicine, Affiliated Hospital of Jiangsu University, Zhenjiang, Jiangsu, China; ^3^ Department of General Surgery, Affiliated Hospital of Jiangsu University, Institute of Digestive Diseases, Jiangsu University, Zhenjiang, Jiangsu, China

**Keywords:** bile acids receptors, bile acid synthesis, bile acid metabolism, macrophage polarization, IBD

## Abstract

Many factors, including genetic vulnerability, barrier function, intestinal immune cells, and intestinal microbiota, may combine to affect the occurrence and progression of inflammatory bowel disease (IBD). Through targeting bile acid receptors (BARs), bile acids have been demonstrated to have a range of regulatory effects on intestinal immune responses in recent decades. As the basis of intestinal immunity, macrophages play an indispensable role in intestinal homeostasis. BARs connect the intestinal microbiota with immune cells, significantly impacting IBD. This review focuses on the role of bile acids in regulating the differentiation and function of intestinal macrophages in IBD.

## 1 Introduction

Although it has long been thought that intestinal microecological abnormalities are connected to several intestinal diseases, including IBD, it is still unclear how the host and intestinal microbiome interact. One significant mechanism of interaction between the microbiome and the host is the production of small molecules by the intermediate or final products of microbial metabolism. As an important component of the metabolism of the intestinal microbiota, bile acids are also signaling molecules that participate in regulating various physiological processes through bile acid receptors (BARs) (1). They have been found to have functions of regulating energy metabolism, inflammatory responses, and immune regulation in metabolic diseases, inflammatory diseases, and tumors (2). Bile acids can be involved in the progression of IBD by participating in the regulation of macrophage recruitment, maturation, polarization and function (3–6). As an important factor connecting the intestinal microbiota and intestinal immune cells, it has become a therapeutic target for exploring new therapies for IBD (7–9). Therefore, we reviewed the metabolic process of bile acids and their role in nutrient absorption, analyzed their regulatory mechanism on macrophages and their role in IBD, and summarized some drug treatment methods for IBD.

## 2 Bile acid metabolism

Bile acids, the primary metabolites of cholesterol, are synthesized at a rate of approximately 1-1.5g per day in healthy individuals. The liver transforms 0.4–0.6g of this into bile acids, which are then expelled with bile (10, 11). Considering their origin, bile acids can be classified as primary and secondary varieties (10). Primary bile acids, such as cholic acid (CA) and chenodeoxycholic acid (CDCA), are synthesized from cholesterol in liver cells and may bind with glycine or taurine. The secondary bile acids, including lithocholic acid (LCA) and deoxycholic acid (DCA),are formed through the deoxygenation of the 7th α hydroxyl group by intestinal bacteria. These acids, along with their conjugates, are combined with glycine or taurine in the liver (12–14). Bile acids are categorized into free and conjugated forms based on their structure. Free bile acid includes CA, CDCA, DCA and a small amount of LCA. These free bile acids combine with glycine or taurine respectively to form various conjugated bile acids including glycocholic acid (GC), taurocholic acid (TCA), glycochenodeoxycholic acid (GCDCA) and taurochenodeoxy cholic acid (TCDCA). Conjugated bile acids are more water-soluble and generally exist in the body as sodium salts, which is more stable than free bile acids.

**Figure 1:**
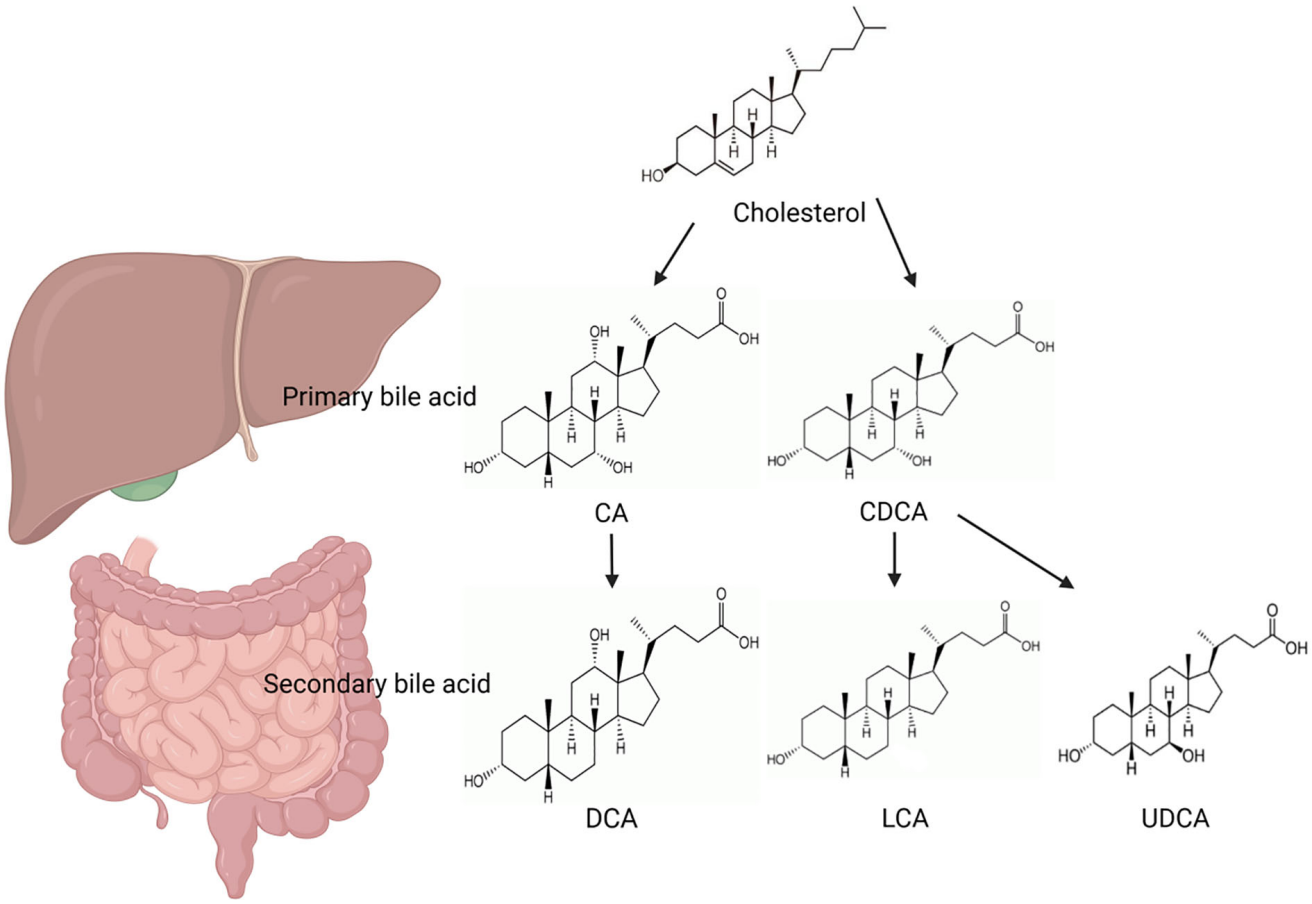
The biosynthetic pathways of primary and secondary bile acids.

There are two types of bile acid synthesis: the classical pathway and the alternative pathway (**Figure 1**) (10, 15, 16). About 90% of primary bile acids are synthesized through classical pathways (10, 13), cholesterol is first catalyzed by cholesterol 7α-hydroxylase (CYP7A1) to form 7α-hydroxycholesterol (10). The conversion of the latter to bile acid includes the 3-αand 12-α hydroxylation, hydroreduction, side chain oxidative cleavage, water addition and other multi-step complex enzymatic reactions, which first produce 24-carbon cholanoyl CoA. The latter can be hydrolyzed to produce primary free bile acids, namely cholic acid and deoxycholic acid, or can be directly combined with glycine or taurine to produce corresponding primary bound bile acids, which are carried into the intestine by bile in the form of sodium or potassium bile acids. Cholesterol 7α-hydroxylase is a key enzyme in the bile acid synthesis pathway and is regulated by the negative feedback of the end product bile acid. After the primary bile acids enter the intestine to promote the digestion and absorption of lipids, secondary bile acids are formed in the ileum and upper colon by the intestinal bacteriase to catalyze the unbinding reaction of bile acids and the dehydroxy effect of 7α (17). Cholic acid removes 7α-hydroxyl to form deoxycholic acid. Chenodeoxycholic removes 7-α hydroxyl to form Lithocholic acid (18). These two free secondary bile acids can also be reabsorbed into the liver through enterohepatic circulation and combined with glycine or taurine to form binding secondary bile acids. In addition, enterobacteria can also convert chenodeoxycholic acid into ursodeoxycholic acid, that is, 7α-hydroxyl of cholic acid into 7β-hydroxyl, which is also classified as secondary bile acid (19). Ursodeoxycholic acid content is very small, although not important for metabolism, but has a certain pharmacological effect. Ursodeoxycholic acid has anti-oxidative stress effect in the treatment of chronic liver disease, can reduce liver damage caused by bile acid retention in the liver, improve liver function and slow down the disease process. The alternative pathway first converts cholesterol to 27-hydroxycholesterol by cholesterol 27-hydroxylase (CYP27A1), then hydroxylated by oxysterol 7-α-hydroxylase (CYP7B1), and then side chain modification to produce CDCA. Bile acids are primarily reabsorbed in the ileum and returned to the liver via portal circulation, where they are released back into the bile, after the promotion of emulsification, digestion, and absorption of lipids and fat-soluble vitamins. Enterohepatic circulation of bile acids is the term for this process (10, 20). About 5% (0.4-0.6g) of bile acids are excreted in the stool, which is in balance with the amount of bile acids synthesized by liver cells.

## 3 The function of bile acids

After being converted into bile by liver cells, bile acids are retained in the gallbladder and released into the intestinal lumen when the gallbladder contracts. Bile acids are crucial for preserving the dissolved state of cholesterol and can aid in the digestion and absorption of lipids. The structure of bile acid is characterized by the three-dimensional configuration of two sides of hydrophilic and hydrophobic structures. This structure makes bile acid have strong interfacial activity and is a strong emulsifier, which can effectively reduce the interfacial tension of oil/water two phases, make the lipid emulsification into 3-10μm fine micro-groups, increase the contact area of lipase and lipase, and facilitate the digestion of fat. Bile acids and the digested lipids will combine to produce phospholipids, which will then form mixed microclusters with a diameter of just around 20 μm., which is conducive to the absorption of lipids through the intestinal epithelial mucosa. 99% of the cholesterol in the human body is excreted through the intestines with bile, of which about two-thirds is excreted in direct form. Cholesterol is difficult to dissolve in water, but cholesterol can form soluble microclusters under the synergic action of bile acid and lecithin, and be transported to the intestine through the biliary tract. The relative concentration of bile acids is an important factor in maintaining the solubility of cholesterol in bile (21),once the balance is broken, cholesterol is easy to precipitate from the bile, forming gallstones, and according to the cholesterol content of gallstones, gallstones can be divided into three categories: cholesterol stones, melanin stones and brown stone (22, 23).

By connecting to bile acid receptors, bile acids can play a variety of roles in controlling immune cell function in addition to their crucial involvement in nutrition absorption and cholesterol dissolution (24). Before the discovery of bile acid receptors, bile acid metabolites CDCA and UDCA had been used as drugs for the treatment of gallstones, which could effectively promote the dissolution of gallstones, and were the first choice for the treatment of gallstones. Today, UDCA remains the primary drug used to alleviate disease progression in primary cholangitis (PBC). In 1999, bile acid receptors were reported (1, 25), the study of bile acids has gained renewed interest. Bile acids are currently believed to primarily function through bile acid receptors, which is a crucial mechanism of action between intestinal immune cells and intestinal microbiota. Investigating intestinal disorders and their processes greatly benefits from the presence of bile acids in conjunction with intestinal microbiota and intestinal immune cells (18).

The two primary categories of bile acid receptors now exist are membrane receptors and nuclear receptors (1, 11, 26). By controlling immune cell activity and bile acid metabolism, it contributes significantly to human health. With the discovery of specific receptors activated by bile acids in intestinal flora, more focus has been placed on how bile acids affect intestinal immune cells. As the first line of defense against bacteria and antigens, intestinal macrophages mediate inflammatory reactions to food, bacteria, and metabolites. Macrophages are regarded as therapeutic targets for a number of illnesses, including IBD, due to their ability to coordinate tissue repair and inflammation resolution (27). According to pertinent research, bile acids can control macrophage polarization via bile acid receptors and are crucial in intestinal disorders (**Table 1**). The development and course of intestinal illnesses are also significantly influenced by their interaction with macrophages. According to recent research, bile acids have two roles in intestinal disorders: they can prevent cancer and reduce inflammation by blocking bile acid receptors, but they can also cause damage that is both carcinogenic and pro-inflammatory. The polarization and function of macrophages may be connected to the various roles.

**Table 1:** BARs and their endogenous bile acid ligands and synthetic ligands.

Cell membrance receptors	Natural bile acid agonists rank of potency	Synthetic ligands
GPBAR1(TGR5)	LCA>DCA>CDCA>UDCA>CA	BAR501,BAR502,INT-767,INT-777
S1PR2	Conjugated bile acid	JTE-013
Nuclear receptors		
FXR	CDCA>DCA>LCA>CA	BAR502; Fexaramine D,GW4064
VDR	LCA and its derivatives	

## 4 Intestinal macrophages

**Figure 2:**
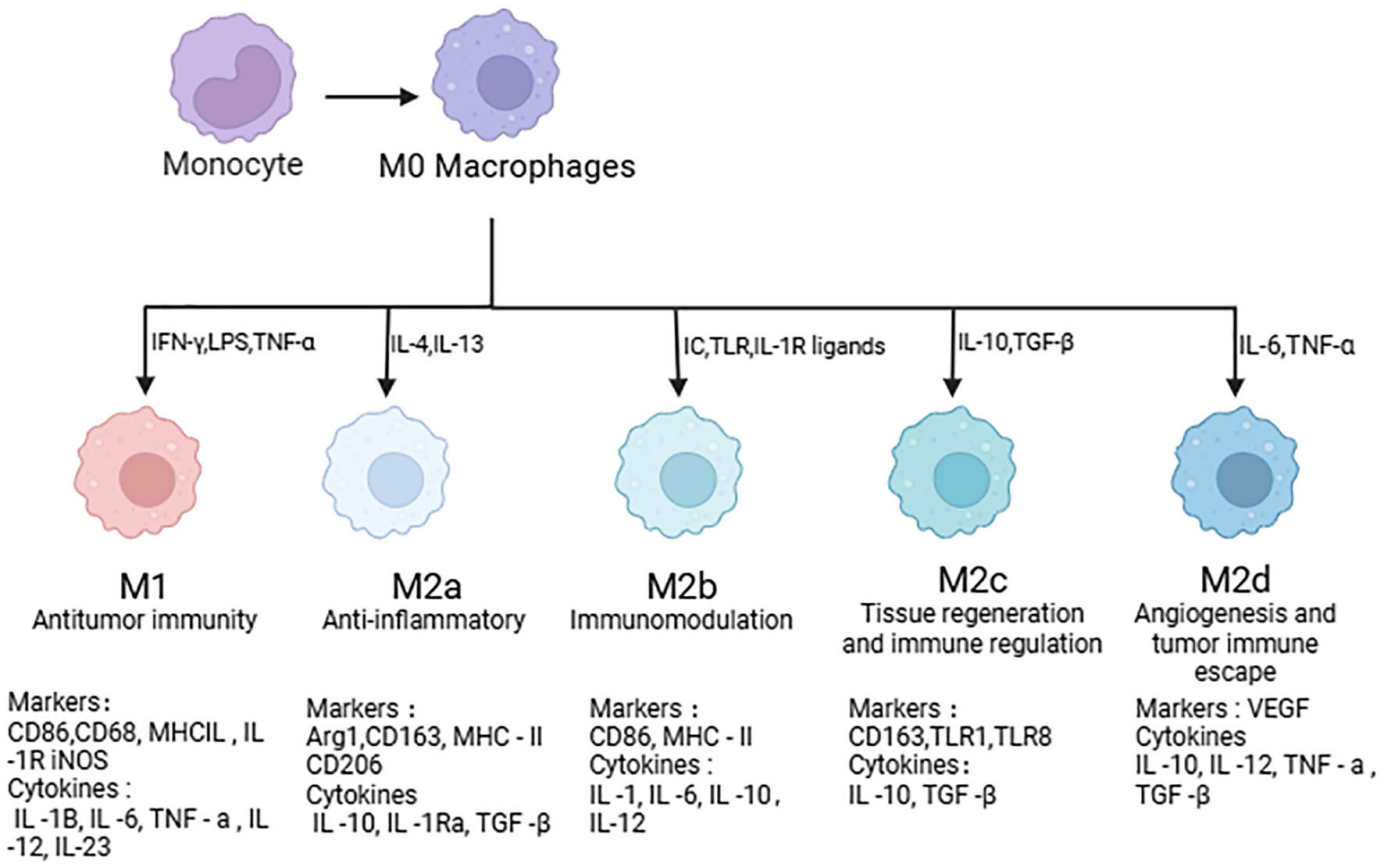
Macrophage polarization.

With the development of the technology, studies have confirmed that most tissue-resident macrophages originate from erythrocyte ⁃ myeloid progenitor cells in the yolk sac during embryonic development, which can regulate function and differentiation according to niche signals, and have the ability to self-renew (28, 29). Macrophages are highly plastic, and the process of producing specific phenotypic and functional responses to microenvironmental stimuli and signals in tissues is called polarization of macrophages. Based on their phenotype and function, macrophages have historically been divided into two types: classically activated macrophages (M1) and alternatively activated macrophages (M2) (30–32) (**Figure 2**).

M1 macrophages are pro-inflammatory cells that, in response to infection or inflammation, release cytokines like interleukins (IL-1β, IL-6, IL-12, and IL-23) and tumor necrosis factor α (TNF-α).It mainly plays the role of promoting inflammation and causing tissue damage (33, 34).

M2 macrophages can be induced into distinct subclasses (M2a, M2b, M2c, and M2d) by various stimulus signals, and they can also have distinct functions in inflammation (35, 36). In addition to expressing elevated amounts of CD206, IL-1 receptor, and CCL17, M2a macrophages can be activated by IL-4 and IL-13.They also release profibrotic substances, encourage Th cell activation, suppress inflammation, support tissue repair, and stimulate angiogenesis (35, 37). M2b macrophages secrete significant amounts of IL-10 and little levels of IL-12, as well as pro-inflammatory cytokines including IL-1β and TNF, when they are triggered by lipopolysaccharide, immune complexes, TLR agonists, or IL-1 receptor ligands (35, 38). Immune complexes, glucocorticoids, prostaglandins, and IL-10 activate M2c macrophages, which then use exudation, extracellular matrix (ECM) remodeling, and angiogenesis to aid in tissue repair (35, 39). Numerous factors, such as co-cultivation with cancer cell ascites or exposure to IL-6, leukemia inhibitors, or purine adenosine, can polarize M2d macrophages (tumor-associated macrophages), which in turn promotes angiogenesis and cancer dissemination (40, 41).

The cause of IBD remains unidentified, but macrophage polarization is pivotal in its immune mechanism. During the active phase of IBD, macrophages polarize towards M1 macrophages and release a large number of inflammatory factors, leading to the continuous progression of intestinal inflammatory response. In the remission period of IBD, M1 macrophages decreased, and their ability to release inflammatory factors was also significantly decreased, and the related markers of M2 macrophages were up-regulated, thus alleviating intestinal inflammatory response. Thus, controlling the polarization and activity of macrophages may aid in alleviating IBD symptoms.

## 5 Bile acid receptors and their functions in macrophages

### 5.1 FXR

Farnesoid X receptor(FXR)is a ligand-dependent bile acid nuclear receptor (BAR), first discovered by Forman in 1995 and named for its transcriptional activity that can be enhanced by physiological concentrations of farnesol (42). In 1999, it was discovered that bile at the physiological level is an endogenous ligand of FXR, hence FXR is known as the BA receptor. It is found throughout the body in many organs, mostly existed in the intestines and liver, and primarily triggered by primary bile acids (42, 43). It is one of the two bile acid-related receptors that have received the most attention. Apart from playing a crucial role in maintaining the balance of bile acids (44, 45), FXR influences the course of numerous illnesses, such as liver cancer, metabolic diseases, gastrointestinal problems, and non-alcoholic steatohepatitis (46–48). Some studies believe that FXR can participate in the maintenance of intestinal homeostasis through various mechanisms, and it can improve intestinal inflammation and inhibit the growth of colitis-related tumors by regulating the recruitment and polarization of intestinal macrophages and crosstalk with Th17 cells. Patients with colitis-associated colorectal cancer (CAC) and IBD had significantly reduced FXR.

FXR is a major regulator of BAs homeostasis, a key gene that regulates bile acid synthesis, transport and reabsorption metabolism (10, 49). FXR mainly regulates bile acid metabolic homeostasis through the liver FXR/SHP axis and ileal FGF 15/19 (FGF19 in humans and FGF15 in mice)/liver FGF receptor 4(FGFR4) axis, and inhibits the activity of rater limiting enzyme CYP7A1,thus inhibiting bile acid synthesis (50–55). After the ileal cells release it, FGF15/19 reaches the liver via portal circulation. In hepatocytes, FGF15/19 binds to FGF-R4, inhibiting CYP7A1 activity and thereby decreasing BAs synthesis in liver tissue (50, 53–55). Bile acids can also up-regulate the expression of SHP protein by activating the FXR-SHP axis, and the up-regulated SHP binds to liver receptor homologous 1 (LRH-1) into heterodimer and inactivates LRH-1, which can positively regulate the expression of CYP7A1 (10).Consequently, the FXR-SHP axis activation suppresses the synthesis of bile acid rate-limiting enzymes, thereby inhibiting bile acid production and maintaining metabolic homeostasis (44, 51, 52). By controlling gene expression to stop bile acid accumulation, FXR protects the liver from bile acids’ damaging effects.

**Figure 3:**
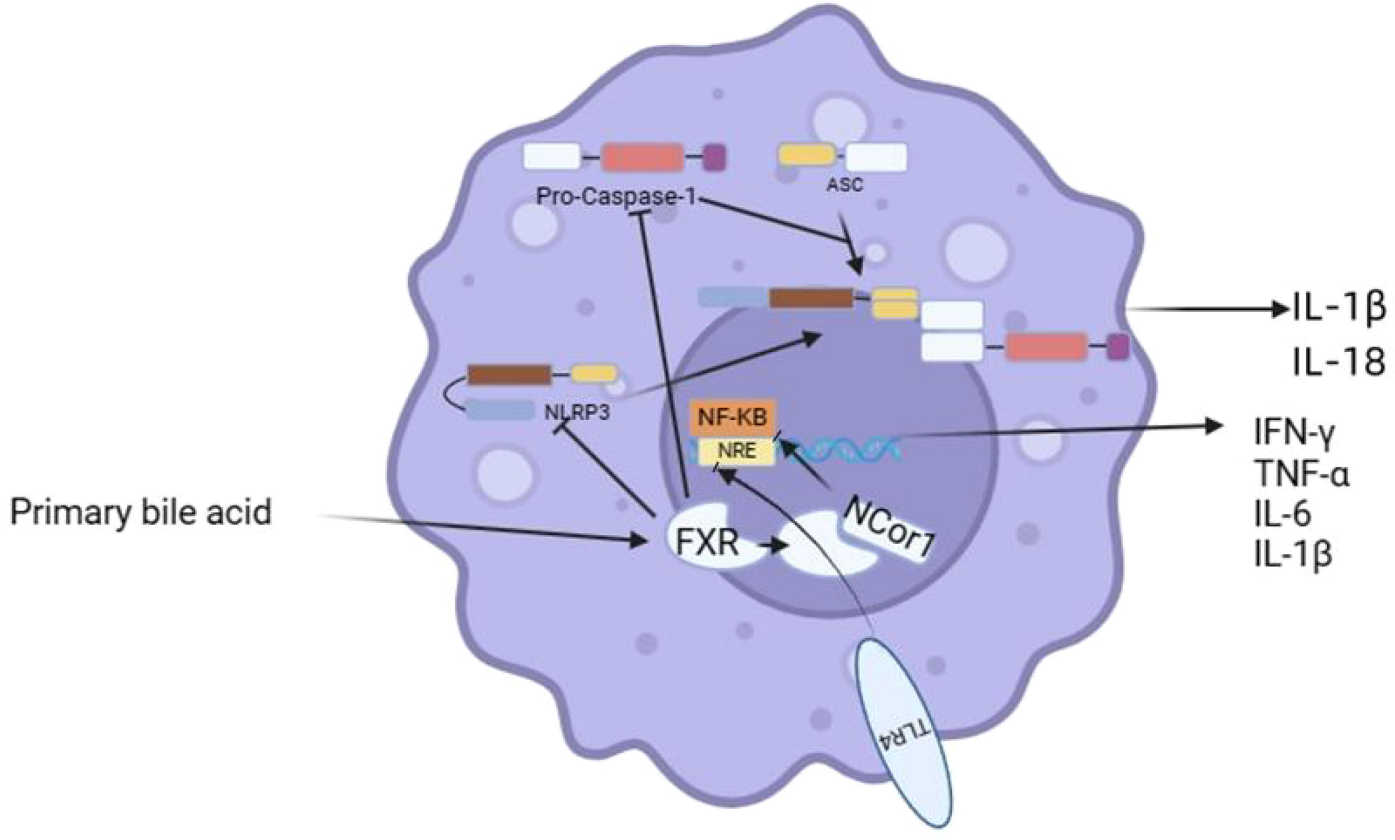
FXR regulates macrophages.

IBD causes intestinal damage and exposes macrophages to more bile acids. FXR stabilizes the nuclear receptor corepressor protein 1 (NCor1) complex by directly targeting the promoters of pro-inflammatory genes, including iNOS, TNF-α, and IL-1β, when it is activated by ligands such as primary bile acids. The NCoR1 complex attaches to gene promoters, blocking NF-kB binding and thus inhibiting inflammatory factor secretion to exert an anti-inflammatory effect. NCoR1 is eliminated from these promoters upon TLR-4 activation. One of the mechanisms by which FXR exerts anti-inflammatory effects in macrophages is through SHP, where FXR regulates SHP in a promotor-dependent manner and blocks the AP-1 pathway by inducing up-regulation of SHP, thereby preventing its binding to inflammatory genes. FXR exerts anti-inflammatory effects by regulating the NLRP3 inflammasome, whose overactivation is linked to various inflammatory diseases. FXR prevents NLRP3 and caspase 1 from physically interacting to activate the NLRP3 inflammasome, which stops them from assembling into an inflammasome (**Figure 3**) (56–58). Many studies have demonstrated that in wild mice with drug-induced colitis, the injection of FXR agonists may considerably reduce intestinal inflammation, block the reduction of cup cells, repair the damaged intestinal mucosal barrier, reduce intestinal permeability, and therefore ameliorate colitis. However, the application of FXR agonists in whole-body FXR knockout mice did not improve colitis. FXR signals are damaged in both IBD and CAC mouse models as well as in IBD and CAC patients. The absence of FXR will increase the susceptibility to inflammation and cancer. Moreover, FXR contributes to the metabolism of carbohydrates and lipids. By encouraging the expression of genes linked to fatty acid oxidation, thermogenesis and mitochondrial biogenesis, FXR activation can help reduce obesity and stop diet-induced weight gain. It is also associated with Browning of adipose tissue and can reduce inflammatory cytokine levels while upregulating beta-adrenergic signaling. FGF15/19 efficiently increases insulin sensitivity and is currently utilized to treat major metabolic illnesses such as diabetes, obesity, and non-alcoholic steatohepatitis (NASH). Activation of FXR can also result in liver insulin sensitization and ameliorate insulin resistance (59). The therapeutic role of Obticholic acid (OCA), an FXR receptor agonist used to treat primary biliary cholangitis (PBC), in hepatic steatosis and cirrhosis has been extensively studied.

Therefore, activating FXR can not only directly affect the differentiation and function of intestinal macrophages and reduce inflammatory factors, but also regulate the recruitment of intestinal macrophages, inhibit the activation and recruitment of intestinal macrophages, and thereby suppress the progression of inflammatory bowel disease (5). In addition to improving intestinal inflammation through intestinal macrophages, it can also regulate bile acid metabolism to restore intestinal homeostasis, and plays an important role in regulating lipopolysaccharide metabolism and energy metabolism. FXR may become an important target for the treatment of IBD and maintaining human health.

### 5.2 GPBAR1

Initially identified by Maruyama et al. in 2002, GPBAR1 is a seven-transmembrane G-protein-coupled receptor that is also referred to as M-BAR or TGR5 (60, 61). GPBAR1 is broadly distributed throughout the body. High levels of GPBAR1 mRNA have been detected in the small intestine, stomach, liver, lung, placenta, spleen, and other organs (62). Secondary bile acids are primarily responsible for its activation, and different types of BAs have different levels of activation on GPBAR1: LCA > DCA > CDCA > UDCA > CA. The physiological ligands of GPBAR1 are the secondary bile acids DCA and LCA (61).

**Figure 4:**
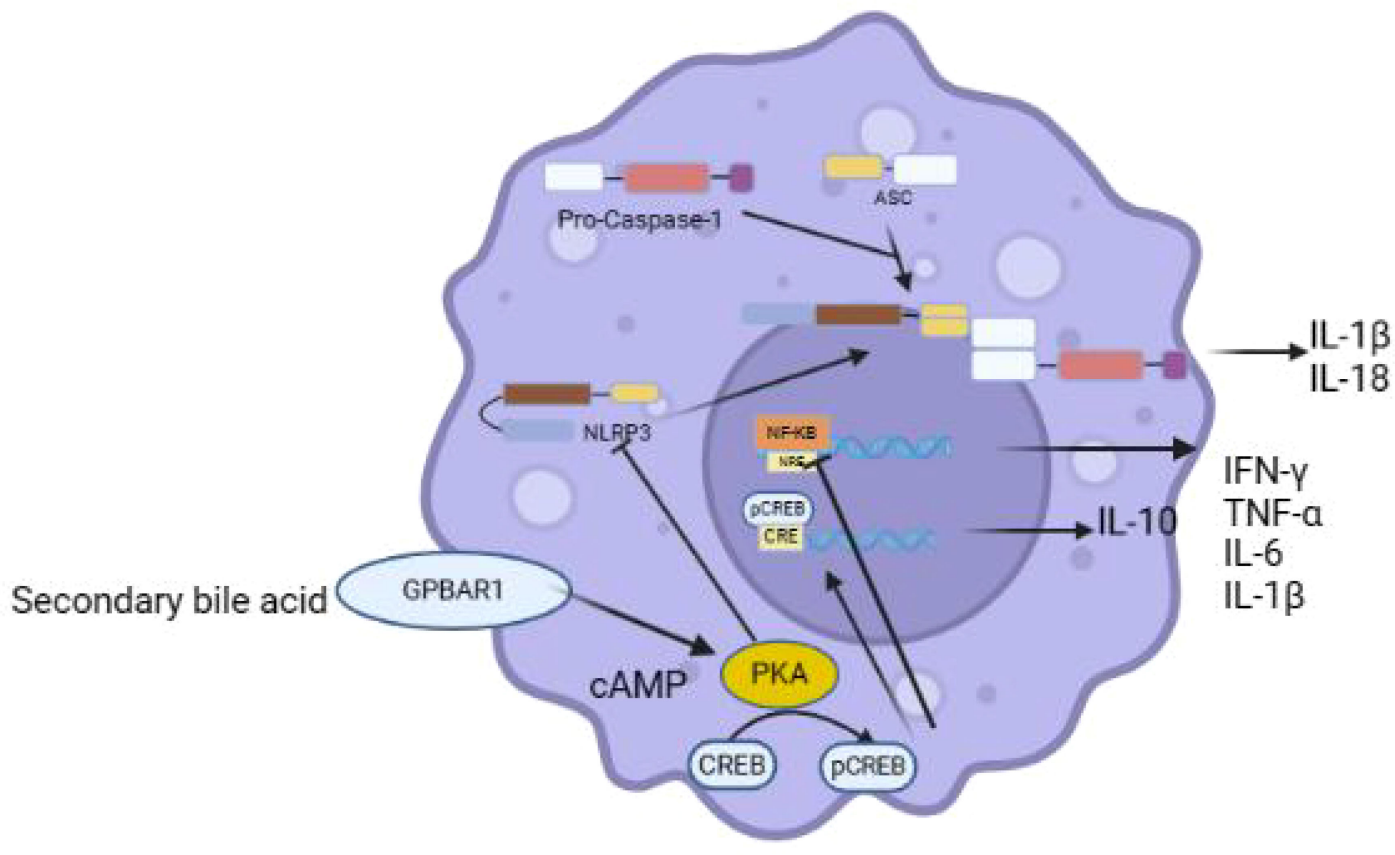
GPBAR1 regulates macrophages.

GPBAR1 is thought to be essential for the preservation of intestine and hepatic immunological homeostasis and is expressed in innate immune cells, macrophages, and NKT cells (62–67). GPBAR1 plays an important role in cell signal transduction. In macrophages, by being activated by DCA and LCA, GPBAR1 increases the amount of cAMP by enhancing the recruitment of cAMP response element binding protein (CREB) to the target gene CRE (cAMP response element), thereby controlling the expression of numerous genes in the target cells. Studies by Michele Biagioli found that under physiological conditions, GPBAR1 activation inhibits the development of inflammatory immune responses and promotes the formation of anti-inflammatory phenotypes in macrophages. This effect may be mediated by the cAMP-response element binding protein (CREB) binding to the IL-10 gene promoter (68, 69). The CAMP-KA-CREb pathway reduces NF-κB activity and inhibits IL-10 secretion, and the use of BAR501, a selective GPBAR1 agonist, promotes IL-10 production by increasing CREB binding to IL-10 promoters, which exerts anti-inflammatory effects (64). By stimulating GPBAR1, the secondary bile acids DCA and LCA can prevent the NLRP3 inflammasome from activating (56, 70). DCA and LCA can lead to the ubiquitination of NLRP3 inflammasome-dependent GPBAR1/cyclic adenosine phosphate (cAMP)/protein kinase A (PKA) pathway, thereby inhibiting its activation and effectively inhibiting the production of IL-1β, and significantly damaging the phagocytosis and secretion functions of macrophages, thus playing a role in inhibiting inflammation (**Figure 4**) (71). Apart from its pro-inflammatory function, IL-1β also encourages the drying of epithelial villi cells, which leads to the development of inflammation-related colon cancers. Furthermore, the activation of GPBAR1 increases the levels of camp-dependent thyroid hormone-activating enzyme (D2-type thyroginine deiodinase) in brown adipose tissue and skeletal muscle cells, promotes the release of glucagon-like peptide-1 (GLP-1), and participates in regulating intestinal motility. These findings collectively indicate that this receptor can be used to treat a variety of metabolic disorders, such as obesity, metabolic syndrome and type 2 diabetes (72–75).

In summary, GPBAR1 promotes anti-inflammatory effects by regulating the polarization of macrophages towards the anti-inflammatory M2 phenotype and inhibiting the production of pro-inflammatory cytokines, thereby maintaining intestinal homeostasis. The activation of GPBAR1 can also enhance the intestinal barrier function and promote the secretion of glucagon-like peptide-1 (GLP-1), which is beneficial to intestinal integrity and metabolic health (76).

### 5.3 VDR

VDR mediates the biological activity of 1,25(OH)2D3 and belongs to the nuclear receptor superfamily (77, 78). The vitamin D-VDR endocrine system is present in almost all nucleated cells. The active form of vitamin D, calcitriol, activates the nuclear receptor known as VDR. Calcitriol engages the VDR to regulate calcium and phosphate levels essential for human homeostasis. VDR can be activated by LCA and its metabolites (78–80). VDR can regulate various diseases of the gut, kidneys, bones, skin, heart, and various other organs (80). For example, the absence of specific VDR in the breast epithelium significantly inhibits pubertal mammary gland development. Lack of VDR in the lungs of mice can lead to early onset of COPD/emphysema, accompanied by chronic inflammatory responses, immune dysregulation, and lung destruction. By decreasing oxidative stress and blocking the autophagy and apoptosis pathways of cardiomyocytes, VDR activation guards against cardiac ischemia/reperfusion injury.

**Figure 5:**
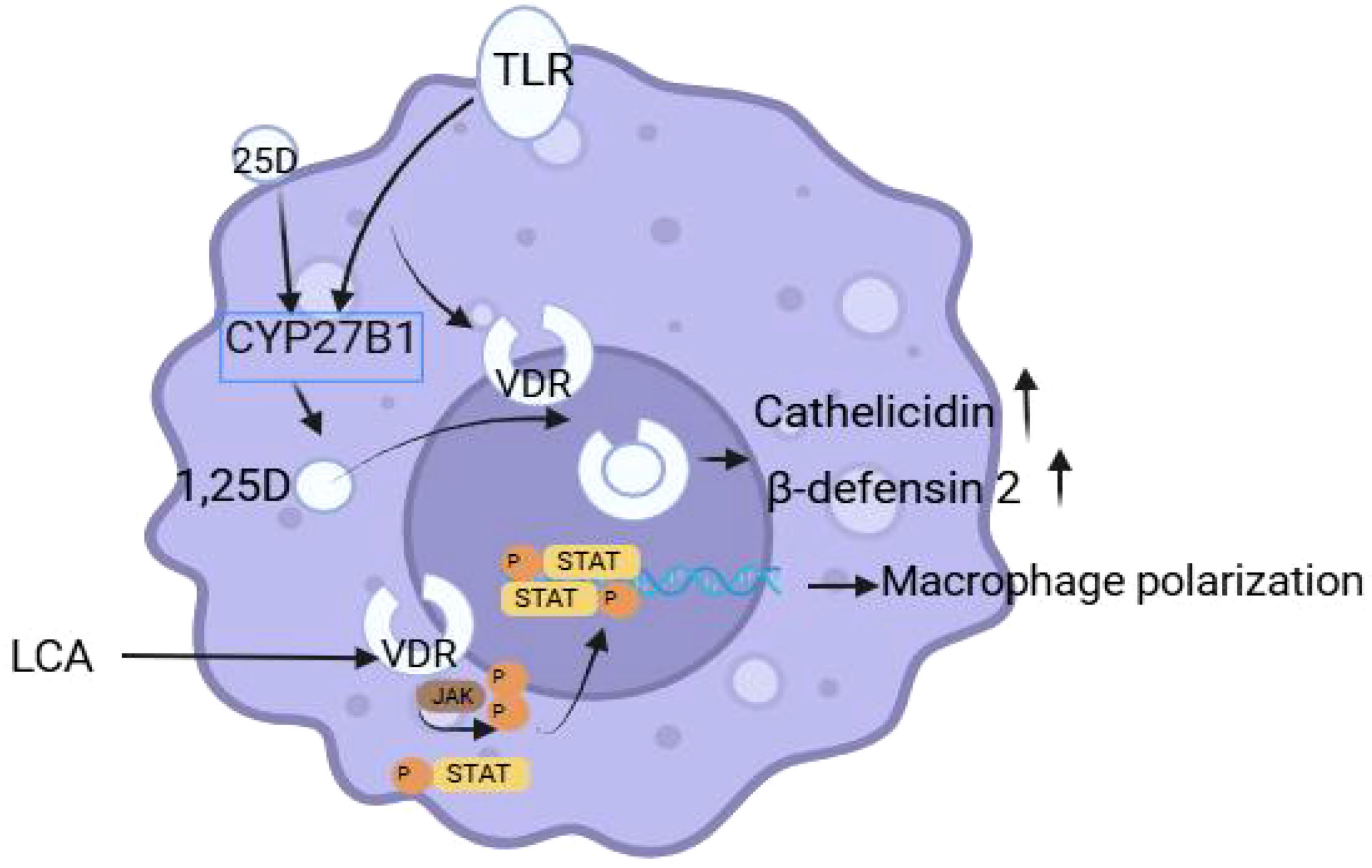
VDR regulates macrophages.

VDR is primarily studied as a bile acid sensor in the gut and is widely expressed in the gastrointestinal tract (81, 82). Its protective effect against colitis and colon cancer has been established (83). Numerous immune cells have the VDR/JAK/STAT signaling pathway, which controls cell activity and has significant implications for improving illness. Previous research indicated that toll-like receptors on monocytes and macrophages detect bacterial, viral, and fungal components, leading to increased expression of the vitamin D receptor (VDR) and CYP27B1 (84). Activation of VDR results in its heterodimerization with the retinoid X receptor (RXR). Heterodimers interact with DNA to stimulate the production of antibiotic peptides, leading to antibiotic-like effects (85, 86). Then, using mice with deficient vitamin D receptor (VDR) in colonic epithelial cells (CEC-VDRKO) or non-intestinal epithelial cells (NEC-VDRKO), the study found that activation of vitamin D receptor can reduce the symptoms and inflammation of colitis and promote the repair of intestinal tissue. 1, 25-dihydroxyvitamin D had anti-inflammatory effects by directly inhibiting M1-type macrophage polarization and promoting M2-type macrophage polarization in mice treated with DSS (**Figure 5**) (87, 88). Through its modulation of the JAK/STAT signaling pathway, VDR can affect the growth of tumors. However, Lu et al. found that Monotropein can regulate macrophage M1-type polarization via VDR/JAK1/STAT1 and inhibit coliti-related cancers (89), it is still necessary to investigate the molecular mechanism of VDR. VDR also regulates the composition and function of gut bacterial communities. Oral 1,25(OH)2D3 supplementation has an effect on the gut microbiota of the human digestive tract, reducing opportunistic pathogens and increasing bacterial abundance.

### 5.4 S1PR2

S1PR2 is a G-protein-coupled receptor, which is activated by the bioactive lipid sphingosine 1-phosphate (S1P), and has various functions such as participating in metabolism, regulating muscle function, and regulating immune cell transport (90–94). Conjugated bile acids with taurine or glycine can also activate S1PR2. Hepatocytes, sinusoidal endothelial cells, bile duct cells, hepatic stellate cells, and macrophages all express S1PR2.

There are five subtypes of the G-protein-coupled receptor S1PR: S1PR1, S1PR2, S1PR3, S1PR4, and S1PR5. S1PR1 signals coordinate the transformation of macrophages into M2 type and the conversion of macrophages into M1 type is regulated by S1PR3 (95–97). By altering signaling pathways and enzymes, S1PR has been shown in numerous studies to have a significant effect on the movement of immune cells, tumor cells and so on.S1PR controls the body’s relative reaction in this manner. Consequently, S1PR has been identified as a potential target for treating autoimmune, lung, liver diseases, and cancer (98).

**Figure 6:**
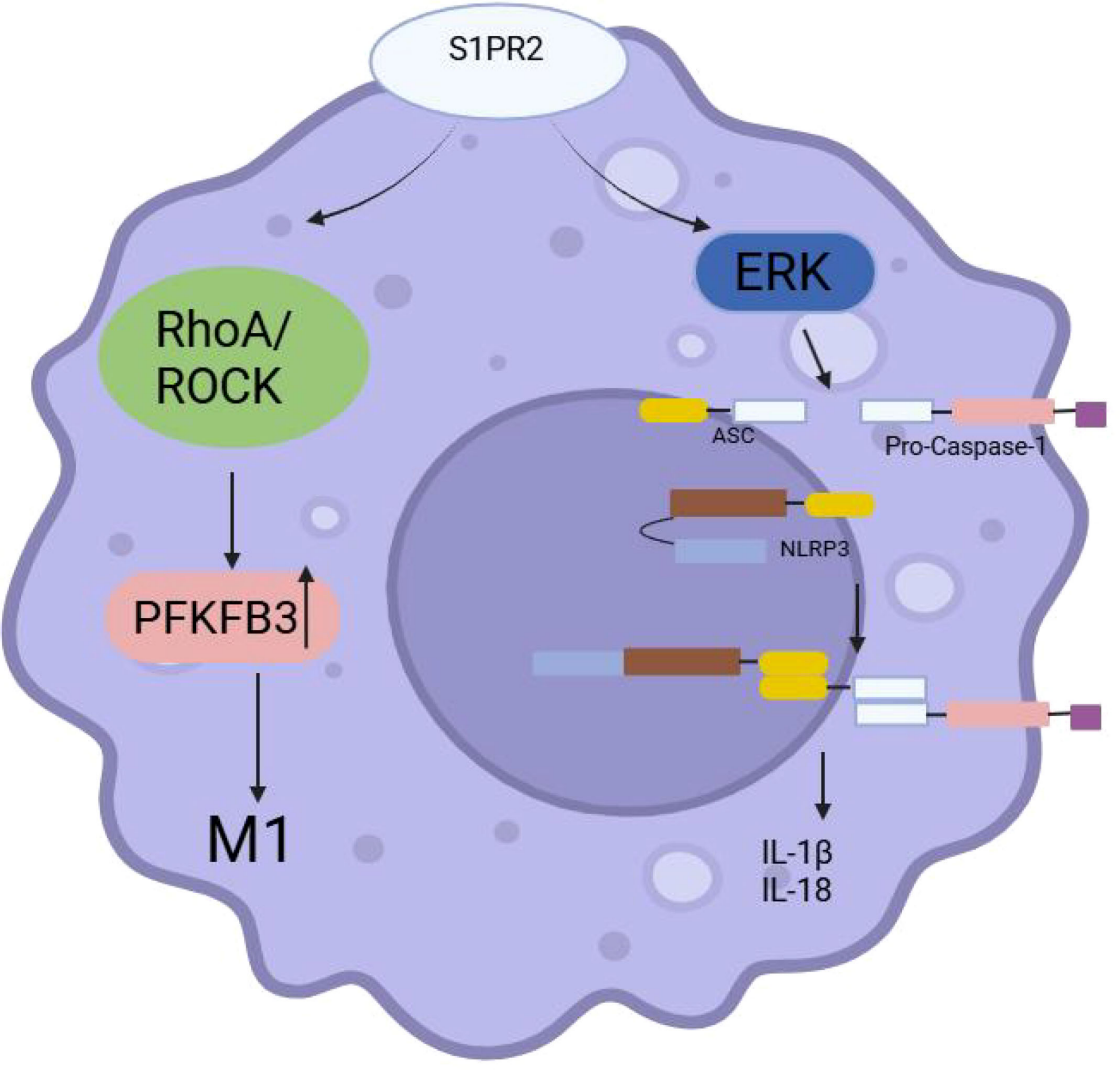
S1PR2 regulates macrophages.

Both intestinal mucosal macrophages from IBD patients and mice with DSS-induced colitis, as well as LPS-treated macrophages *in vitro*, showed markedly increased expression of S1PR2.The S1PR2/RhoA/ROCK1 signaling pathway potentially contributes to IBD development by influencing M1 macrophage polarization (99). Additionally, S1PR2 is a significant target for IBD treatment due to its functions in regulating vascular permeability, immune cell transport, and preserving intestinal epithelial barrier integrity. Research indicates that S1PR2-/-deficient mice exhibit heightened sensitivity to dextran sulfate-induced colitis, characterized by increased intestinal permeability and CD4+ T cell proliferation (100). Another study showed that neither the inhibition of FXR nor the knockdown of TGR5 expression in a high-fat diet had a notable effect on the production of DCA-promoted proinflammatory cytokine IL-1β. According to the study, DCA induces the release of pepsin B, at least partially, through S1PR2, which in turn activates the NLRP3 inflammasome (**Figure 6**). This, in turn, causes the macrophages to produce IL-1β in a dose-dependent manner. Given that colitis-associated cancer (CAC) is closely linked to persistent, uncontrolled intestinal inflammation, this may also be another possible mechanism by which secondary bile acid DCA is involved in colon cancer development.

## 6 Treatment

The global incidence of IBD has been rising annually, predominantly affecting young individuals. Most IBD patients have repeated attacks and are not cured, and inflammatory bowel disease is progressive. A considerable number of patients need surgical treatment due to complications, such as ileostomy, subtotal colectomy or total colectomy. If left untreated, it may lead to a series of irreversible long-term complications, such as colitis-related colorectal cancer (101). With the development of science and technology, the treatment of IBD has kept pace with The Times. In the early 20th century, colitis treatment primarily involved bed rest and enemas. After 1930, the surgical treatment of UC has made great progress, and ileostomy, subtotal colectomy or total colectomy are still retained (102). The discovery of sulfonamides and antibiotics contributed to the emergence of the first effective UC treatment drugs, and subsequently, glucocorticoids, immunosuppressants and other drugs were found to be successfully used in the treatment of IBD, and the introduction of anti-tumor necrosis factor α (TNF-α) monoclonal antibodies made the treatment of IBD enter the era of biological agents. Currently, IBD treatment drugs are categorized into para-aminosalicylic acid, glucocorticoids, immunosuppressants, biologics, and small molecule drugs (**Table 2**).

**Table 2:** Drug therapy for inflammatory bowel disease.

Type	Medicine	Characteristics	Common adverse reactions	Precaution	References
Para-aminosalicylic acid	Sulfasalazine	Colon release	Gastrointestinal reactions, rash, headache, reversible male infertility, allergic reactions, hepatotoxicity, hematological toxicity, aseptic pneumonia	Not for use in children under 2 years old, as it has many drug interactions, including with oral hypoglycemic agents and oral anticoagulants.	Kirsner JB (103), Ko CW (104)
	Mesalazine	Ileum and colon release	Diarrhea, nausea and headache	Combined use with adrenaline and anticoagulants increases the tendency to bleed and enhances the hypoglycemic effect of sulfonylureas.	
	Olsalazine	Release at distal jejunum,ileum and colon	Diarrhea, nausea and headache	Not for use in patients with severe liver or kidney dysfunction.	
Glucocorticoid	Budesonide	The doses of other types of systemic action hormones are converted based on the dose of prednisone	Hypercortical syndrome induces or aggravates infection, osteoporosis, muscle atrophy, and delayed wound healing	Gradually reduce the dosage until the medication is stopped as the symptoms ease. It cannot be used for maintenance treatment.	Kadmiel M et al. (105)
Immunosuppressant	Tacrolimus, Thalidomide	Suitable for those who are ineffective or dependent on glucocorticoid	Gastrointestinal reactions, hepatotoxicity, infection, bone marrow suppression, and pancreatitis	For patients with hormone-dependent UC, a low dose of [1.3mg/(kg.d)] azathioprine can effectively maintain disease remission; Thalidomide is suitable for the treatment of refractory UC	Lichtiger S (106), Singh S (107)
Biologics and small molecule drugs	Adalimumab, certolizumab pegol, golimumab	Glucocorticoid and the above-mentioned immunosuppressants are ineffective or lucocorticoi -dependent or intolerant to the above-mentioned drugs	Infection, gastrointestinal discomfort, allergies, headaches, malignant tumors	Contraindications: ① Active infection, chronic infection or a history of repeated infections in the recent period; ② Congestive heart failure ③ Malignant tumors (including current symptoms and past medical history); ④ Demyelinating lesions of the nervous system; ⑤ Allergic to murine protein components; ⑥ Pregnancy period.	Peyrin-Biroulet L (108)

The active ingredient in aminosalicylic acid, 5 aminosalicylic acid (5 ASA), reduces inflammation and antioxidant to treat IBD. In addition, 5 ASA can regulate the immunity and correct the imbalance of intestinal flora in UC patients, and play a role in preventing colon cancer (109). Among them, sulfasalazine (SASP) is the first drug discovered, and its therapeutic effect on UC and active CD has been confirmed in a number of studies (103, 110, 111). Only a small fraction of SASP is absorbed in the small intestine. Most of the drugs are decomposed by bacteria after reaching the colon and release 5 ASA and SP. The former plays a therapeutic role locally in the colon mucosa, while the latter acts as an inert carrier to ensure the release of 5 ASA in the colon. The side effects of SASP include gastrointestinal reactions, rash, headache, reversible male infertility, allergic reactions, hepatotoxicity, hematological toxicity, aseptic pneumonia, etc. The occurrence of these adverse reactions is considered to be closely related to the blood concentration of SP, so the development of new preparations of 5-ASA without SP to reduce adverse reactions is the focus of research (112).

Cytoplasmic glucocorticoid receptor (GR) can regulate the transcription of anti-inflammatory protein genes by binding to glucocorticoids. This process inhibits pro-inflammatory gene activation and promotes the degradation of their mRNA, resulting in significant anti-inflammatory effects (105). As early as the 1950s, glucocorticoids were found to have a positive effect on symptom improvement in UC patients. The dose, manner, and duration of administration all affect the likelihood and intensity of the majority of glucocorticoid-related adverse effects (113). At the same time, hormone resistance is another concern in hormone therapy (114).

Immunosuppressants are the third class of drugs introduced for the treatment of IBD, but the initial indications of this class of drugs do not include IBD. With the development and widespread clinical application of biologics, the positioning of immunosuppressants in the treatment of IBD has changed, and the combination treatment with biologics may be more effective and beneficial, but more evidence is needed.6-mercaptopurine(6-MP) and azathioprine(AZA) have been shown to be effective and relatively safe in the induction and maintenance of remission of IBD, postoperative maintenance therapy of CD, and chemopprophylaxis of colorectal cancer (115–117). Hepatotoxicity, infection, pancreatitis, bone marrow suppression, and gastrointestinal problems are among the common side effects of AZA and 6-MP. Long-term use may raise the chance of developing cancerous growths such skin cancer and lymphoma (118).

Biologics and small molecule drugs can bind to specific targets to effectively control clinical symptoms and disease progression in patients with IBD by blocking downstream inflammatory responses and lymphocyte migration. The first biologics introduced for IBD treatment were anti-TNF-α drugs. Common adverse reactions of biologics and small molecule drugs include infection, gastrointestinal discomfort, allergy, headache, etc. Common serious adverse events include severe infection, opportunistic infection, and malignant tumor. The production of anti-antibodies are closely related to the loss of response to biologic agents and allergy, and combined immunosuppressive therapy can significantly reduce the production of anti-antibodies (119). The development of medications that target bile acid receptors to treat cholestasis and metabolic disorders has advanced recently (46, 47). Currently, various bile acid receptor ligands have demonstrated potential in experimentally treating diseases such as IBD. A derivative of the anthocyanode oxycholic acid, obticholic acid (OCA) is an FXR agonist, has been utilized in clinical studies to treat NASH. The intestinal environment’s stability is maintained via the JAK/STAT-mediated signaling system. A novel oral JAK inhibitor called tofacitinib has been approved in clinical studies to treat UC and may potentially be useful in treating CD (120). Tofacitinib decreased intestinal inflammatory response in UC patients by inhibiting M1 macrophage polarization, which in turn decreased the production of inflammatory markers. Meanwhile, *in vitro* studies indicate that vitamin D influences macrophage polarization, warranting further investigation into its role as an immunomodulator in IBD treatment.

## 7 Conclusions and perspectives

Maintaining bile acid homeostasis in life is crucial since studies conducted in recent decades have shown how vital bile acids are for immunity. The importance of bile acids interacting with the gut microbiome is well understood and extensively studied.

In addition to their role in lipid metabolism, bile acids have become important metabolites of pleiotropic signals as important regulators of intestinal immune system and intestinal microbiota. Bile acids can regulate the development and function of intestinal immune cells by acting as natural ligands on bile acid receptors that exist in these cells. This impacts the gut’s immunological homeostasis. In this review, the latest advances in bile acid metabolism and bile acid receptor regulation of intestinal macrophages are reviewed. Although important advances have been made in past studies, many important questions remain. Due to its low specificity, bile acids can bind to various bile acid receptors and have immunomodulatory effects. Bile acid receptors are widely distributed in the body and exist in various tissues and organs. Secondly, due to the cytotoxic effects of bile acids and their metabolites themselves, the use of bile acids in the treatment of IBD still requires further research. The intestinal microbiota, intestinal immune cells and intestinal microenvironment interact and influence each other, making it still difficult to use bile acids in the treatment of IBD. The search for new, safe and effective targeted drugs is the focus of the next research. As mentioned in the article, some targeted drugs have been proven to have good therapeutic effects on inflammatory bowel disease. Some of the latest studies also indicate that bile acid receptor dual agonists have therapeutic potential in IBD models. Dual-target drugs or multi-target drugs, especially those with synergistic effects, will have greater advantages in improving therapeutic efficacy and reducing drug resistance. In conclusion, although there are still some difficulties in the treatment of IBD with bile acid receptors, there is strong evidence supporting the role of bile acid receptors in regulating macrophages and IBD. Discovering and validating brand-new dual-target or multi-target drugs and developing highly specific targeted drugs is one of the main development directions in the future, which has significant clinical significance.
